# Coordinated Dispersion and Aggregation of Gold Nanorod in Aptamer-Mediated Gestational Hypertension Analysis

**DOI:** 10.1155/2019/5676159

**Published:** 2019-11-11

**Authors:** Xiucui Bao, Gaoxiang Huo, Li Li, Xuebin Cao, Yamei Liu, Thangavel Lakshmipriya, Yeng Chen, Firdaus Hariri, Subash C. B. Gopinath

**Affiliations:** ^1^Department of Obstetrics, Yihe Maternity District of Cangzhou People's Hospital, Cangzhou, Hebei 061000, China; ^2^Department of General Surgery, Cangxian Hospital, Cangzhou, Hebei 061000, China; ^3^Institute of Nano Electronic Engineering, Universiti Malaysia Perlis, 01000 Kangar, Perlis, Malaysia; ^4^Department of Oral & Craniofacial Sciences, Faculty of Dentistry, University of Malaya, 50603 Kuala Lumpur, Malaysia; ^5^Department of Oral and Maxillofacial Clinical Sciences, Faculty of Dentistry, University of Malaya, 50603 Kuala Lumpur, Malaysia; ^6^School of Bioprocess Engineering, Universiti Malaysia Perlis, 02600 Arau, Perlis, Malaysia

## Abstract

Gestational hypertension is one of the complicated disorders during pregnancy; it causes the significant risks, such as placental abruption, neonatal deaths, and maternal deaths. Hypertension is also responsible for the metabolic and cardiovascular issues to the mother after the years of pregnancy. Identifying and treating gestational hypertension during pregnancy by a suitable biomarker is mandatory for the healthy mother and foetus development. Cortisol has been found as a steroid hormone that is secreted by the adrenal gland and plays a pivotal role in gestational hypertension. A normal circulating level of cortisol is involved in the regulation of blood pressure, and it is necessary to monitor the changes in the level of cortisol during pregnancy. In this work, aptamer-based colorimetric assay is demonstrated as a model with gold nanorod to quantify the level of cortisol using the coordinated aggregation (at 500 mM of NaCl) and dispersion (with 10 *μ*M of aptamer), evidenced by the scanning electron microscopy observation and UV-visible spectroscopy analysis. This colorimetric assay is an easier visual detection and reached the limit of detection of cortisol at 0.25 mg/mL. This method is reliable to identify the condition of gestational hypertension during the pregnancy period.

## 1. Introduction

Gestational hypertension or pregnancy-induced hypertension complicates ∼10% of the pregnant cases and causes a poor perinatal outcome. It is also responsible for raising other diseases, such as elevated blood pressure in the artery, preeclampsia, and eclampsia, during the period of pregnancy [1, 2]. In addition, there is a possibility of affecting other parts in the body, such as kidney and heart, and inducing an early delivery. In general, gestational hypertension arises during the second half of pregnancy. Identifying hypertension by a suitable biomarker is mandatory for a healthy pregnant woman [3]. Cortisol is a stress hormone that is secreted from the adrenal gland. It spikes into the main stream of the body during the time of high stress and elevates the cortisol level in the bloodstream. It has been proved that the serum cortisol plays a major role in the pathophysiology of the gestational hypertension [4], especially the higher level of cortisol causes hypertension and endothelial dysfunction [5]. 11*β*-Hydroxysteroid dehydrogenase type 2 (11*β*-HSD2) is an enzyme, produced in the renal tubules; it converts cortisol into an inactive cortisone, thus permitting the mineralocorticoid (a receptor) as aldosterone-selective. The functional diminishes of this enzyme cause the mutations with *HSD11B2* gene, which encodes 11*β*-HSD2, mainly considered as an initiative for hypertension [6]. Therefore, measuring the level of cortisol of the pregnant women during the trimester is considered to be important for estimating the function of 11*β*-HSD2 [7].

In the current investigation, an aptamer-based colorimetric assay was performed to quantify the level of cortisol. Aptamer, a DNA or RNA molecule, has been generated from the randomized library of molecules by a method “SELEX” (Systematic Evaluation of Ligands by Exponential enrichment) with three vital steps, which includes binding, separation, and amplification [8–11]. Since aptamers carry the advantages over antibodies, such as easier to synthesize, cheaper, amenable to the modifications, high affinity, and nonimmunogenic, various aptamers were generated against a wide range of targets from the lower-molecular weight molecules to the intact cell. The generated aptamers have been applied in different fields such as medical, environmental, drug delivery, imaging, and biosensors. Due to the highly selective and sensitive binding nature of aptamer to its target molecule, it has been widely applied in the field of biosensors and more prevalent to diagnose various diseases at a higher affinity. Aptamers that were demonstrated with various biosensors, which include surface plasmon resonance [12, 13], waveguide mode sensor [14], colorimetric [15], and RAMAN spectroscopy [16], help to detect diseases from the basic viral infection to death-causing diseases, such as cancer [17–20]. Among the revealed sensors, colorimetric analysis with aptamers brings out several positive features, such as easier visualization, rapidness, cheaper, effective, and used to detect tiny analytes including heavy metals [21], smaller molecular weight proteins [22], DNA [23], and cancer biomarkers [24], without involving sophisticated instrumentation and trained personnel [25].

The visual colorimetric analysis is the salt-induced aggregation assay by utilizing DNA, RNA, or aptamers with the gold nanostructure to detect the desired target. Gold is one of the unavoidable materials in the field of biosensors due to its versatile physical and chemical properties. Moreover, gold nanoparticle (GNP) is smaller, suitable to confine the electrons in order to produce the quantum effects, a key consideration for the colorimetric assay [26]. In addition, the functionalized GNP leads to find several downstream applications. Due to the abovementioned positive features, gold nanomaterial and the gold surface have been applied efficiently in all types of sensor to detect different biomarkers [8, 27–30]. In general, the unmodified dispersed GNPs have a bright red-wine color and changes its color to purple or blue when it aggregates under ionic condition [30, 31]. This controlled change in color induced by the aggregation can be the basis of colorimetric assay. In the case of aptamer-based colorimetric assay, aptamers are immobilized on the surface of the gold through the electrostatic attraction [15]. When the aptamer is bound to the target, the color of the GNP solution changed to purple at a high salt concentration. This study has utilized a modified gold nanorod (GNR) attached with anticortisol aptamer to interact with the cortisol (target), a model system that can be applied to measure the gestational hypertension by quantifying the cortisol as in earlier study [32].

## 2. Materials and Methods

### 2.1. Materials

Gold nanorod (GNR) was obtained from Nanocs, USA. Sodium chloride (NaCl) was procured from Sigma-Aldrich, USA. The hormones cortisol and progesterone were from Adooq Biosciences (USA). Norepinephrine was from Abcam (USA). Anticortisol sequence was adapted from Sanghavi et al. [32] and synthesized commercially. Buffers and other reagents were obtained in pure form and used directly. The size and shape of the GNR were observed under field-emission scanning electron microscopy at 500 nm scale.

### 2.2. Optimization of Monovalent Ions on Gold Nanorod for Salt-Induced Aggregation

To perform the colorimetric assay, first optimize a suitable concentration of NaCl to induce the aggregation of GNR. Different concentrations of NaCl were added independently with the constant volume of 10 *μ*l GNR (final concentrations were 15, 30, 60, 125, 250, and 500 mM) and kept for 10 min at room temperature. The changes in the colors were noticed and the maximum wavelength absorbance was measured by using the UV-visible spectrophotometer, in which the scanned wavelength ranged from 400 to 750 nm.

### 2.3. Optimization of Aptamer Attachment on Gold Nanorod

Before performing the colorimetric assay, the condition was optimized for the right aptamer concentration to stabilize the GNR at a high salt concentration. Different concentrations of the diluted aptamer were mixed with 10 *μ*l of GNR (final concentration will be 1.25, 2.5, 5, 10, 15, and 20 *μ*M) independently and kept for 30 min at RT. After that, the optimal higher concentration of NaCl was added to each dilution and incubated for 10 min to observe the changes with the color of GNR. The changes in the colors were noticed and the absorbance wavelength maximum was measured by using the UV-visible spectrophotometer scanned from 400 to 750 nm.

### 2.4. Colorimetric Detection of Cortisol Using Anticortisol Aptamer on GNR

The aptamer modified GNR (aptamer-GNR) was used to detect the cortisol. For that, 1 mg/mL of cortisol was added with aptamer-GNR and kept for 30 min at RT. Then, the higher concentration of NaCl was added to the solution to observe the color change. After the confirmation of detection, to evaluate the limit of detection, the cortisol concentrations were titrated from 0.625 to 1 mg/mL by interacting aptamer-GNR. Specific detection of cortisol was carried out with two control hormones, namely norepinephrine and progesterone. For that, 1 mg/mL of control hormone was mixed with aptamer-GNR and incubated for 30 min at RT. Then, NaCl was added to evaluate the interaction of aptamer with control hormones. The results obtained were compared with 1 mg/mL of cortisol interaction with aptamer-GNR.

## 3. Results and Discussion

Gestational hypertension is a critical disorder during the pregnancy period and it causes various issues with foetus development and delivery. Finding a level of hypertension is necessary to take care of the mother and baby during and after the period of pregnancy. Cortisol is the stress hormone and its level plays a crucial role in causing different diseases, such as gestational hypertension, during pregnancy.

In the materials study, it has been widely accepted that the wavelength shift of the plasmon band with the gold shows a big impact in the biosensing applications. In general, the spherical-shaped gold particles have been used for the colorimetric assay to induce a large shift for the high-performance detection, in which the controlled aggregation and dispersion causes the spectral difference and in the presence of the target, aggregation with ionic solution displays a broad spectrum under UV-visible spectroscopy scanning. However, GNP-based colorimetric assay is not suitable for multiplex analysis due to the absence of a properly shaped spectrum. Researchers are looking for an alternate particle to minimize a wide spectrum in order to move towards the multiple target analysis. It has been revealed that the usage of anisotropic silver nanoparticles with tetrahedron shows a spectral shift upon target interaction but it does not cause the aggregation, and demonstrated a microarray for molecular fingerprint analysis. Researchers also proposed the usage of GNR to overcome the high aggregation, as GNR can be fabricated at different range of size ratios and has unique advantage for multiplex analysis. Towards this direction, the current study is an attempt to optimize the condition for future multiplex analysis [33]. To support this notion, researchers have demonstrated the multiplex detection based on the plasmon changes by GNR [33].

**Figure 1:**
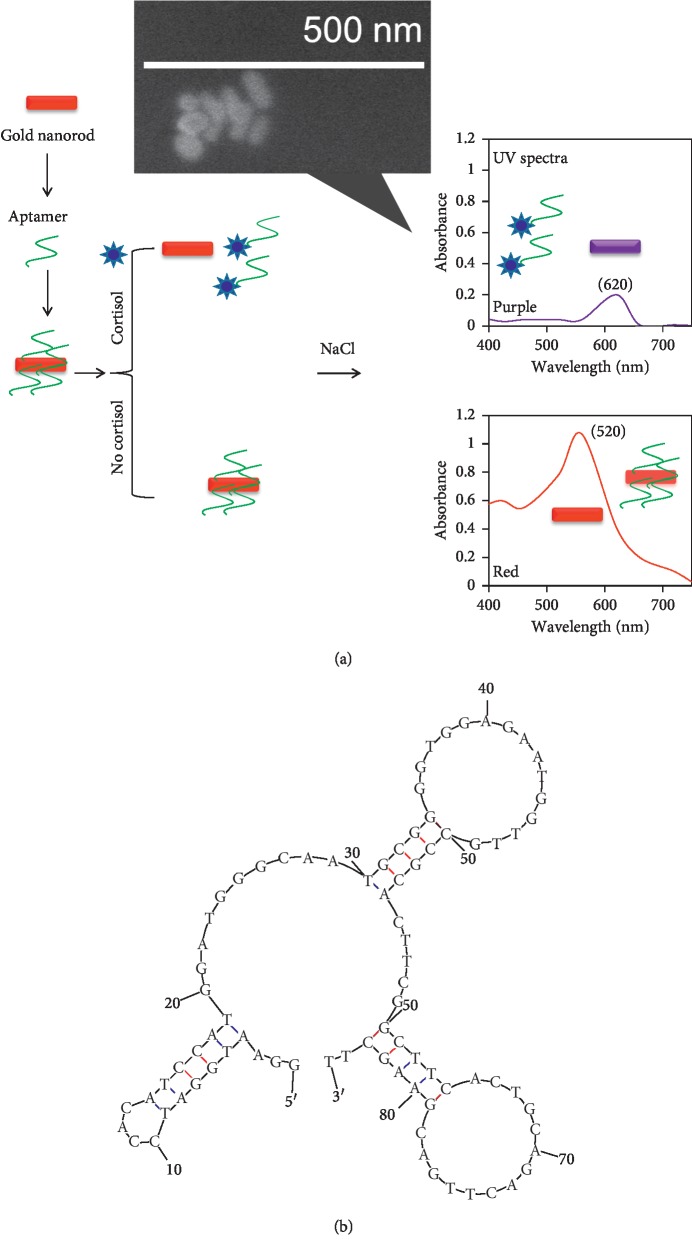
(a) Schematic representation of cortisol detection by aptamer-GNR based colorimetric assay. As-received GNR appears red and in the presence of NaCl, it turned into purple. At higher concentration of aptamer-GNR, it appears to be red even in the presence of NaCl. When aptamer-GNR reacts with cortisol at appropriate concentration, the color of the GNR solution is turned to purple with NaCl, indicating the release of the aptamer from the GNR. The aggregation is displayed by SEM analysis (inset). (b) Secondary structure of anticortisol aptamer. Folded by mfold online software.

To proceed in this line, the current research has been carried out to detect the level of cortisol by a gold nanorod- (GNR-) based aggregation on colorimetric assay using an aptamer generated against cortisol.  Figure 1 shows the schematic representation of the colorimetric assay-based detection of cortisol. As shown in the figure, aptamers are electrostatically bound on the surface of GNR, and upon interacting with cortisol aptamers will be released from GNR. With this condition when adding the higher concentration of NaCl, the GNR will be aggregated and the color of the solution is turned into purple from red (dispersion) (Figure 1(a)). The predicted secondary structure of the tested anticortisol aptamer by mfold software is shown in Figure 1(b), and the aptamer has apparent stems and loops to interact with cortisol.

### 3.1. Requirement of Optimal Monovalent Ion for GNR Aggregation

**Figure 2:**
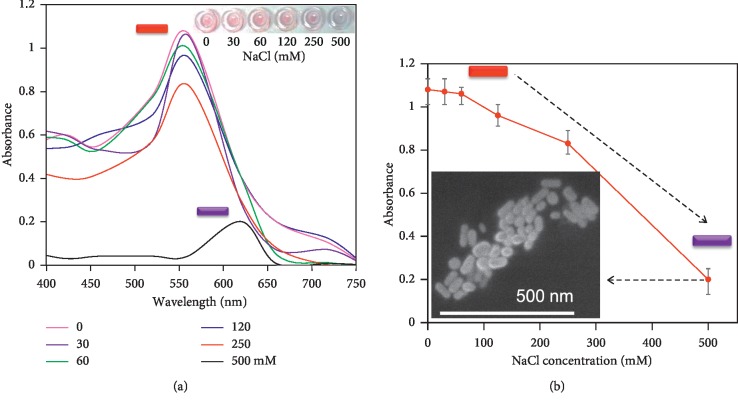
NaCl titration on GNR. (a) Different concentrations of (30 to 500 mM) NaCl were mixed independently with a constant amount of GNR and the aggregation pattern was observed. Inset displays the color developments. (b) Peak absorbance maximum with different concentrations of NaCl, averaged with different experimental replicates. Inset is for aggregation obtained by SEM.

Before initiating the detection of cortisol, determination of a suitable concentration of NaCl is necessary to achieve higher sensitivity; for that, different concentrations of NaCl were tested with the constant GNR volume.  Figure 2(a) displays the obtained UV-visible spectrum with NaCl titration on GNR. It is clearly seen that with increase in the NaCl concentration, the optical density (OD) of the GNR was reduced. With the concentration from 30 to 250 mM, the peak position has not been shifted, but at the higher concentration (500 mM), the apparent peak shift was noticed from 550 to 620 nm, due to the aggregation of GNR (Figure 2(b)). The aggregation was evidenced by the field-emission scanning electron microscopy observation (Figure 2(b), inset). This aggregation is due to the NaCl bridging on the unmodified negatively charged GNRs, resulting in appearance of purple or blue solution [31].

### 3.2. Requirement of Optimal Aptamer Concentration for GNR Dispersion

**Figure 3:**
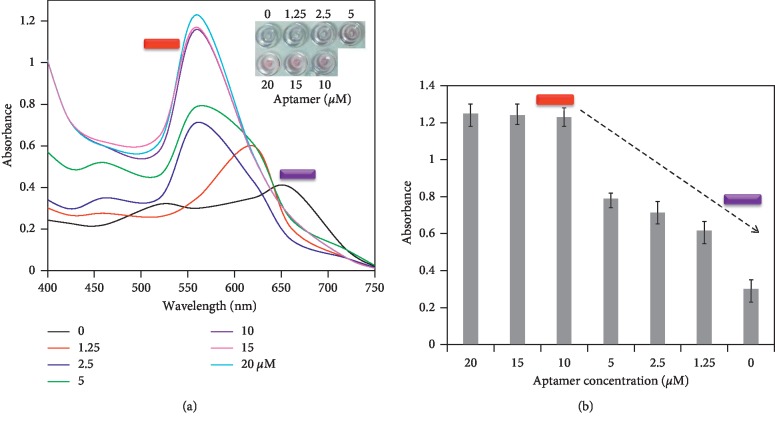
Optimization of aptamer concentration. (a) Aptamer with concentrations of 1.25 to 20 *μ*M was mixed independently with GNR and the aggregation was checked in the presence of NaCl. Inset displays the color developments. (b) Peak absorbance maximums with different concentrations of aptamer, averaged with different experimental replicates. The arrow indicates the direction of the changes.

Upon finding the optimal concentration at 500 mM of NaCl, the experiment was performed to find a suitable concentration of anticortisol aptamer to cover completely the surface of GNR, to be stable under 500 mM of NaCl in the absence of cortisol. Without the complete coverage, the GNR will cause the aggregation by NaCl even in the absence of cortisol and leads to the erroneous positive result. For the optimization analysis, initially different concentrations from 1.25 to 10 *μ*M of aptamer were mixed independently with the fixed volume of GNR and 500 mM (final concentration) NaCl was added to check the stability of GNR. In general, thiol-conjugated aptamers have been used to immobilize them on the surface of the gold and they are very stable under a high salt concentration. In our case, we directly immobilized the unmodified aptamer on the surface of the GNR. In principle, the aptamer or single-stranded DNA can attract to the surface of gold nanostructure due to the coordination between gold and “N” atoms in DNA bases. As shown in Figure 3, the aptamer concentration with 1.25 *μ*M shows the peak maximum at 620 nm with the optical absorbance of 0.6, indicating GNR is in the aggregated form in the presence of NaCl. By increasing the aptamer concentration further, the peak intensity was also increased and all the higher concentrations of aptamer showed the peak at the wavelength ∼550 nm, indicating that GNR is in the dispersal state. At the aptamer concentration of 10 *μ*M, the solution shows the maximum peak intensity with optical absorption as 1.2. This result confirms that the aptamer concentration needed to detect the cortisol ideally by the colorimetric assay is 10 *μ*M. As shown in Figure 3(a), the concentration at 10 *μ*M causes the formation of an apparent peak at 550 nm with a clear change of the solution to red. To confirm that this concentration is the optimum, we performed the experiments with further concentrations at 15 and 20 *μ*M. The results clearly displayed that 10 *μ*M is the optimum concentration for the current colorimetric assay for the cortisol detection (Figures 3(a) and 3(b)).

### 3.3. Genuine Interaction of Cortisol and Nonbiofouling

**Figure 4:**
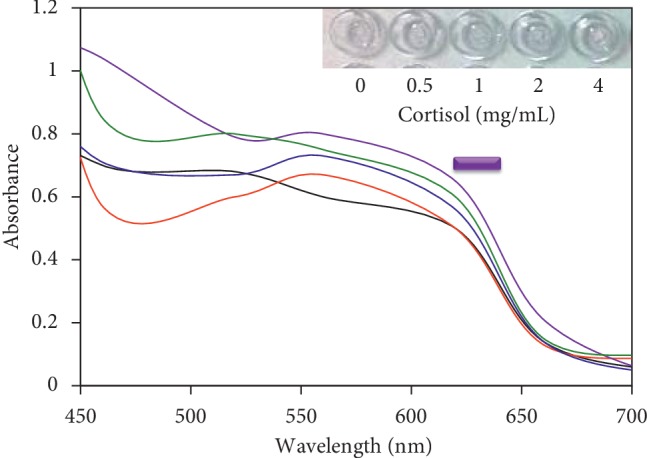
Nonfouling effect of cortisol on GNR. Different concentrations of cortisol (0.5–4 mg/mL) were mixed independently with constant amount of GNR, and 500 mM NaCl was added to evaluate the nonfouling effect.

The abovementioned experiments were used to determine the optimal NaCl and aptamer concentrations. Before proceeding further for the cortisol detection, the nonspecific binding of cortisol on the surface of the GNR was tested. If cortisol itself binds on the GNR, it may lead to a false-negative result. For this analysis, different concentrations of cortisol (0.5, 1, 2, and 4 mg/mL) were mixed independently with the constant GNR and induced the color change by 500 mM of NaCl. It was noticed that even when the concentration of cortisol was increased, the color of the GNR solution turned to purple in the presence of NaCl due to the aggregation, which means that the cortisol itself is not able to bind on the surface of the GNR, and similar results were observed with all the concentrations tested (Figure 4).

### 3.4. Cortisol in Aggregation of Aptamer-GNR

**Figure 5:**
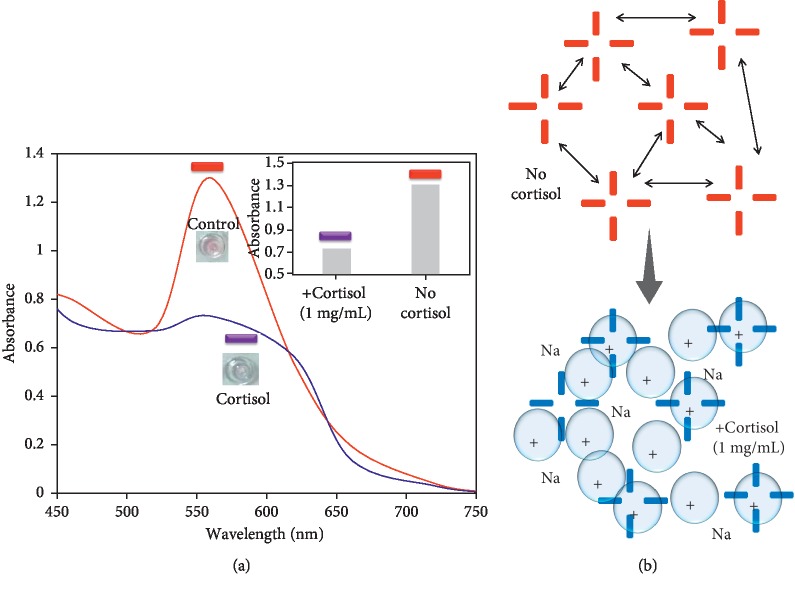
(a) Detection of cortisol by colorimetric assay on aptamer-GNR conjugates. 1 mg/mL of cortisol was mixed with aptamer-GNR and checked the aggregation in the presence of NaCl. Inset displays the graphical representation. (b) Mechanism of dispersion and aggregation mimics the above reaction.

After all the optimizations, we performed the colorimetric assay using aptamer, cortisol, and GNR with the above final conditions. Initially, the higher concentration (1 mg/mL) of cortisol was used to evaluate the release of aptamer from the GNR. As shown in Figure 5, in the control experiment using aptamer-GNR (without cortisol), we did not observe the changes with the spectrum at different wavelengths, and it still remains same at the wavelength 550 nm. This means that in the absence of cortisol, the aptamer-GNR kept its red color under a high concentration of salt. At the same time, when we mixed 1 mg/mL of cortisol to aptamer-GNR, the cortisol interacts with aptamer on GNR and released. Under this condition, at the higher salt concentration, the color of the solution turned into purple and the spectrum was shifted from 550 to 620 nm due to the aggregation (Figure 5(a) and inset). The apparent mechanism with the aggregation and dispersion is shown in the Figure 5(b).

### 3.5. Determination of Limit of Detection with GNR Aggregation

**Figure 6:**
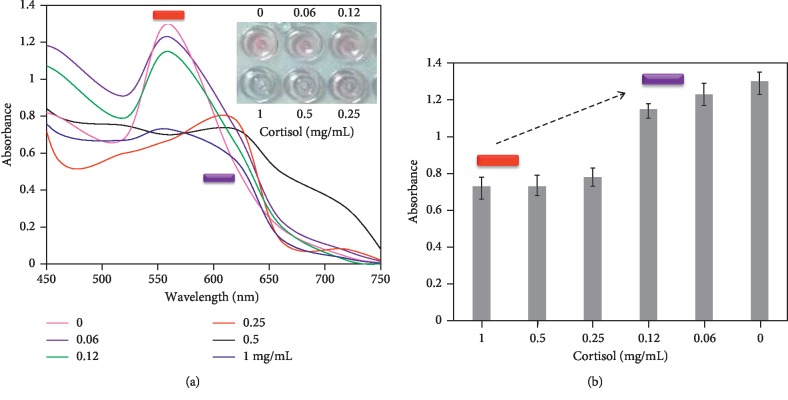
(a) Limit of detection with cortisol. Cortisol concentrations from 0 to 1 mg/mL were mixed independently with GNR-aptamer conjugates and the aggregation was checked in the presence of NaCl. Inset displays the color developments. (b) Peak absorbance maximums with different concentrations of cortisol, averaged with different experimental replicates. The arrow indicates the direction of the changes.

**Figure 7:**
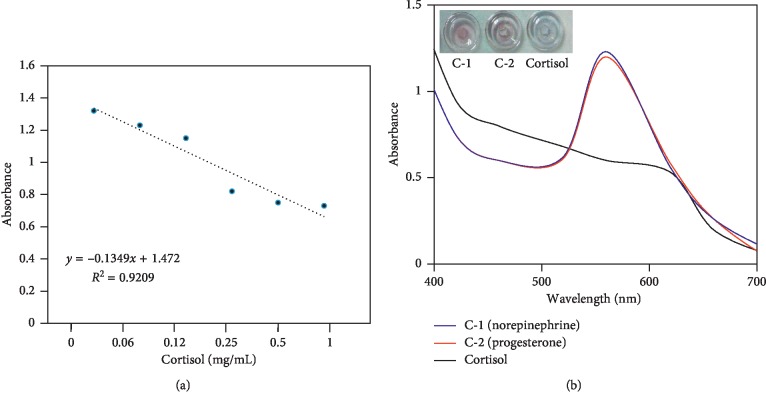
(a) Linear relationship between the absorption maximum and the concentration of the cortisol. 0 to 1 mg/mL of cortisol were used to detect by using GNR-aptamer conjugates. (b) Specific detection of cortisol was carried out with two different control hormones (norepinephrine (C1) and progesterone (C2)). The aptamers interacted with only cortisol, and the color of the GNR was changed to purple due to the aggregation.

Since it was found that 1 mg/mL of cortisol was clearly detected by the colorimetric assay, to evaluate the limit of detection, the titration was performed with the cortisol from 1 mg/mL down to 0.06 mg/mL under similar experimental conditions.  Figure 6 explains the results of the cortisol detection at different concentrations. It was noticed that the color of the solution clearly changed at three concentrations (1, 0.5, and 0.25 mg/mL) of cortisol and the corresponding shifts in optical absorbance were found to be 0.73, 0.73, and 0.78, respectively, at ∼620 nm. The concentrations from 0.12 to 0.06 mg/mL did not show the spectral change and still retain their peak maximums at 550 nm, which indicated that these cortisol concentrations are not sufficient to release the aptamer from GNR.  Figure 7(a) shows the linear relationship between the absorption maximum and the concentration of the cortisol. From these results, it was concluded that the limit of detection was found at 0.25 mg/mL of cortisol using the colorimetric assay and that it is more suitable to monitor the gestational hypertension with the changes in cortisol levels.

### 3.6. Comparative Analysis and Specificity

Table 1 summarizes the quantitative detection of cortisol by different methods including the conventional strategies. The primary advantages of the current colorimetric method is the visual detection by naked eye, which is absent in other methods for cortisol detection. In addition, colorimetric method does not need any prior handling experiences and the special instruments, thereby making it appealing over other methods. Furthermore, to compliment the obtained results, it was compared with the interdigitated electrode sensor and a clear binding was noticed with the same concentration used in the colorimetric assay. However, interdigitated electrode sensor gives a clear response compared to the colorimetric assay (Supplementary information ([Supplementary-material supplementary-material-1])).

Specific detection of cortisol was shown by performing the experiment with two different control hormones namely, norepinephrine and progesterone. As shown in Figure 7(b), with the control hormones, the GNR did not show the changes in color and the absorbance peak maximums were stable at 550 nm. At the same time with the cortisol, the aptamer was released from the GNR upon interaction, and the color of the GNR solution was changed to purple due to the aggregation in the presence of NaCl. From these results, it was concluded that cortisol was specifically detected by the colorimetric assay with aptamer-GNR.

## 4. Conclusion

Gestational hypertension causes various health issues to the mother and baby during and after the period of pregnancy. Identifying the real condition of hypertension with a suitable biomarker is mandatory to treat properly. In this work, cortisol, known as the “stress hormone,” was detected by the colorimetric assay using aptamer and gold nanorod conjugate as the primary tools. Cortisol was clearly detected by showing the color change of the gold nanorod solution turning to purple from red with monovalent salt, and the limit of detection was found as 0.25 mg/mL. This method of detection has advantages over other methods to quantify the levels of cortisol with a higher specificity and helps to treat gestational hypertension.

## Figures and Tables

**Table 1:** Comparison among the available methods for the quantitative cortisol detection.

Method	Material	Probe	Sensitivity	Reference
Chemiresistor	Graphene	Antibody	10 pg/mL	[34]
Integrated electrode	MOS_2_	Antibody	1 ng/mL	[35]
Printed electrode sensor	Graphene	Antibody	0.1 ng/mL	[36]
Electrochemical sensor	Cofired ceramic	Antibody	10 pg/mL	[37]
Electrochemical impedance spectroscopy	Zinc oxide	Antibody	1 ng/mL	[38]
Piezoelectric immunosensor	Gold-coated surface	Antibody	36 *μ*g/mL	[39]
RAMAN spectroscopy	—	Antibody	Human serum (ng/mL)	[40]
Electrochemical impedance spectroscopy	Gold electrode	Antibody	0.5 mg/mL	[41]

## Data Availability

All the data are fully available without restriction.

## Abbreviations

GNR: Gold nanorod
nM: Nanomolar
nm: Nanometer
mM: Millimolar
OD: Optical density
*μ*l: Microliter
11*β*-HSD2: 11*β*-Hydroxysteroid dehydrogenase type 2
SELEX: Systematic Evaluation of Ligands by Exponential Enrichment
GNP: Gold nanoparticle
RNA: Ribonucleic acid
DNA: Deoxyribonucleic acid.

## Supplementary Materials

Supplementary Materials It includes the detection of cortisol on interdigitated electrode sensor, for the comparative study. It also includes brief method and the obtained results. Figure S1: the interaction of cortisol and aptamer on interdigitated electrode sensor. Reference for the described method is also provided.
