# Supplementary material for: Coordinated Dispersion and Aggregation of Gold Nanorod in Aptamer-Mediated Gestational Hypertension Analysis
Source: J Anal Methods Chem. 2019 Nov 11;2019:5676159. doi: 10.1155/2019/5676159 (PMC6881590; doi:10.1155/2019/5676159)
Supplement: Supplementary Materials — It includes the detection of cortisol on interdigitated electrode sensor, for the comparative study. It also includes brief method and the obtained results. Figure S1: the interaction of cortisol and aptamer on interdigitated electrode sensor. Reference for the described method is also provided. [file 5676159.f1.docx]

**Co-ordinated Dispersion and Aggregation of Gold Nanorod in**

**Aptamer-mediated Gestational Hypertension Analysis**

Xiucui Bao^1*^, Gaoxiang Huo^1^, Li Li^1^, Xuebin Cao^2^, Yamei Liu^1^,

Thangavel Lakshmipriya^3^, Yeng Chen^4^, Firdaus Hariri^5^, Subash C.B. Gopinath^3,6^

^1^Department of Obstetrics, Yihe maternity District of Cangzhou People's Hospital, Cangzhou, Hebei, 061000, China.

^2^Department of General Surgery, Cangxian Hospital, Cangzhou, Hebei, 061000, China

^3^Institute of Nano Electronic Engineering, Universiti Malaysia Perlis, 01000 Kangar, Perlis, Malaysia.

^4^Department of Oral & Craniofacial Sciences, Faculty of Dentistry, University of Malaya, 50603 Kuala Lumpur, Malaysia.

^5^Department of Oral and Maxillofacial Clinical Sciences, Faculty of Dentistry, University of Malaya, 50603 Kuala Lumpur, Malaysia.

^6^School of Bioprocess Engineering, Universiti Malaysia Perlis, 02600 Arau, Perlis, Malaysia.

Xiucui Bao: [baoxiucui@sina.com](mailto:baoxiucui@sina.com)

Gaoxiang Huo: [13582711701@sina.cn](mailto:13582711701@sina.cn)

Li Li: [13832761632@sina.cn](mailto:13832761632@sina.cn)

Xuebin Cao: [caoxuebin2018@sina.com](mailto:caoxuebin2018@sina.com)

Yamei Liu: [yamei_0816@sina.com](mailto:yamei_0816@sina.com)

Thangavel Lakshmipriya: lakshmipriya50@gmail.com

Yeng Chen: [chenyeng@um.edu.my](mailto:chenyeng@um.edu.my)

Firdaus Hariri: firdaushariri@um.edu.my

Subash C.B. Gopinath: subash@unimap.edu.my

***Correspondence:** [baoxiucui@sina.com](mailto:baoxiucui@sina.com)

**Detection of cortisol on interdigitated electrode sensor: Comparative study**

The detection of cortisol by gold nanorod-aptamer based colorimetric assay was compared with interdigitated electrode (IDE) electrochemical sensor. For that, the IDE sensing surface was fabricated as described earlier (Iswary et al., 2019) and modified chemically by Carbonyl diimidazole (CDI). A 0.5 M of diluted CDI in 30% ethanol was added on the IDE sensing surface to immobilize the 1 mg/mL of cortisol. And then the remaining surface was blocked by 1 M of ethanolamine. Finally, aptamer-GNR conjugates were added on the surface to check the interaction of cortisol and aptamer. As shown in the figure S1, the black line is the cortisol binding on the CDI modified surface, after added the ethanolamine the current level was increased (red line). And then when the aptamer-GNR was added, the level of the current decreases. This confirms the genuine binding of aptamer and cortisol. This result supports our detection system for binding cortisol with GNR-aptamer conjugates. By comparison, both systems can detect the corticol, however, each carries different advantages. Colorimetric assay is easier, cheaper and no need the prior experiences compared to the IDE sensor. On the other hand, IDE sensor is highly sensitive compared to the colorimetric detection, but need prior experience with a proper training to handle the experiments.

Voltage (v)

Cortisol

Ethanolamine

GNR-aptamer

Current (A)

Figure S1: Interaction of Cortisol and aptamer on interdigitated electrode sensor.

**Reference**: Letchumanan, I., Md Arshad, M.K., Balakrishnan, S.R., Gopinath, S.C.B. 2019. Gold-nanorod Enhances Dielectric Voltammetry Detection of C-reactive protein: A Predictive Strategy for Cardiac Failure. ***Biosensors and Bioelectronics*** 130:40-47.
